# Supplementary material for: Analysis of emergent patterns in crossing flows of pedestrians reveals an invariant of ‘stripe’ formation in human data
Source: PLoS Comput Biol. 2022 Jun 9;18(6):e1010210. doi: 10.1371/journal.pcbi.1010210 (PMC9216623; doi:10.1371/journal.pcbi.1010210)
Supplement: S1 Table — Table summarizes the results of the ANOVA tests that were performed for each α with γ¯, γ˜L and γ˜R to check their statistical dependencies on whole-crowd and separate-group analyses under the pattern matching technique. (PDF) [file pcbi.1010210.s001.pdf]

crossing angle α	F	p	η^2
26.1°	$F(2,50) = 2.004$	0.145	0.074
63.8°	$F(2,51) = 1.147$	0.326	0.043
89.8°	$F(2,54) = 0.808$	0.451	0.029
116.9°	$F(2,48) = 1.642$	0.204	0.06
154.1°	$F(2,48) = 0.377$	0.688	0.015
179.7°	$F(2,48) = 0.207$	0.814	0.008

Table summarizes the results of the ANOVA tests that were performed for each α with $\bar{\gamma}$, $\tilde{\gamma}_L$ and $\tilde{\gamma}_R$ to check their statistical dependencies on whole-group and separate-group analyses under the pattern matching technique.
