# Supplementary material for: Analysis of emergent patterns in crossing flows of pedestrians reveals an invariant of ‘stripe’ formation in human data
Source: PLoS Comput Biol. 2022 Jun 9;18(6):e1010210. doi: 10.1371/journal.pcbi.1010210 (PMC9216623; doi:10.1371/journal.pcbi.1010210)
Supplement: S2 Table — Table summarizes the results of the ANOVA tests that were performed for each α with λ¯, λ˜L and λ˜R to check their statistical dependencies on whole-crowd and separate-group analyses under the pattern matching technique. (PDF) [file pcbi.1010210.s002.pdf]

crossing angle α	F	p	η^2
$26.1°$	$F(2,50) = 0.4$	0.672	0.015
$63.8°$	$F(2,51) = 0.898$	0.414	0.034
$89.8°$	$F(2,54) = 5.832$	0.005	0.177
$116.9°$	$F(2,48) = 0.542$	0.585	0.022
$154.1°$	$F(2,48) = 0.109$	0.897	0.004
$179.7°$	$F(2,48) = 1.362$	0.266	0.054

Table summarizes the results of the ANOVA tests that were performed for each α with $\bar{\lambda}$, $\tilde{\lambda}_L$ and $\tilde{\lambda}_R$ to check their statistical dependencies on whole-group and separate-group analyses under the pattern matching technique.
