# Supplementary figures and images for: Analysis of emergent patterns in crossing flows of pedestrians reveals an invariant of ‘stripe’ formation in human data
Source: PLoS Comput Biol. 2022 Jun 9;18(6):e1010210. doi: 10.1371/journal.pcbi.1010210 (PMC9216623; doi:10.1371/journal.pcbi.1010210)

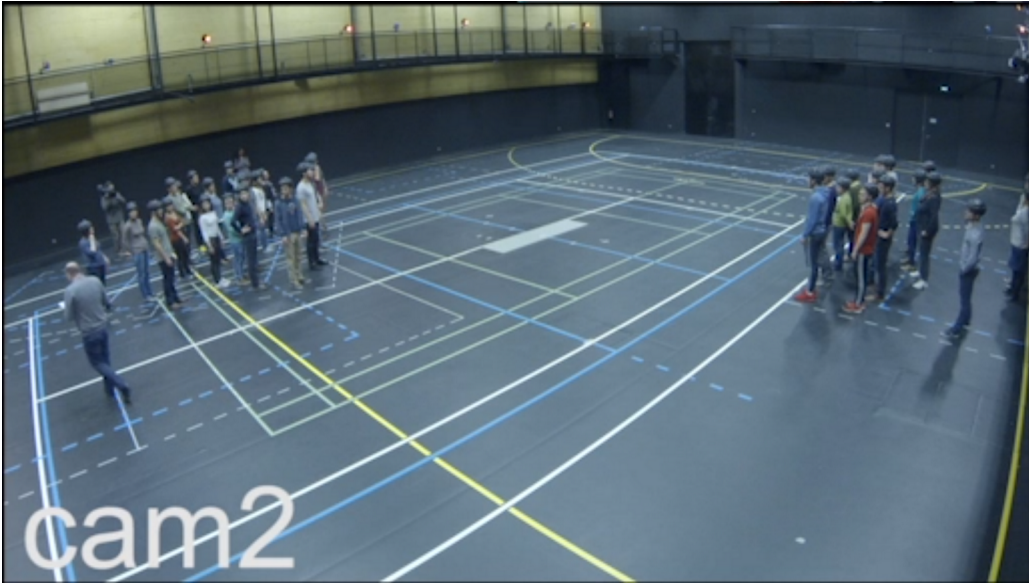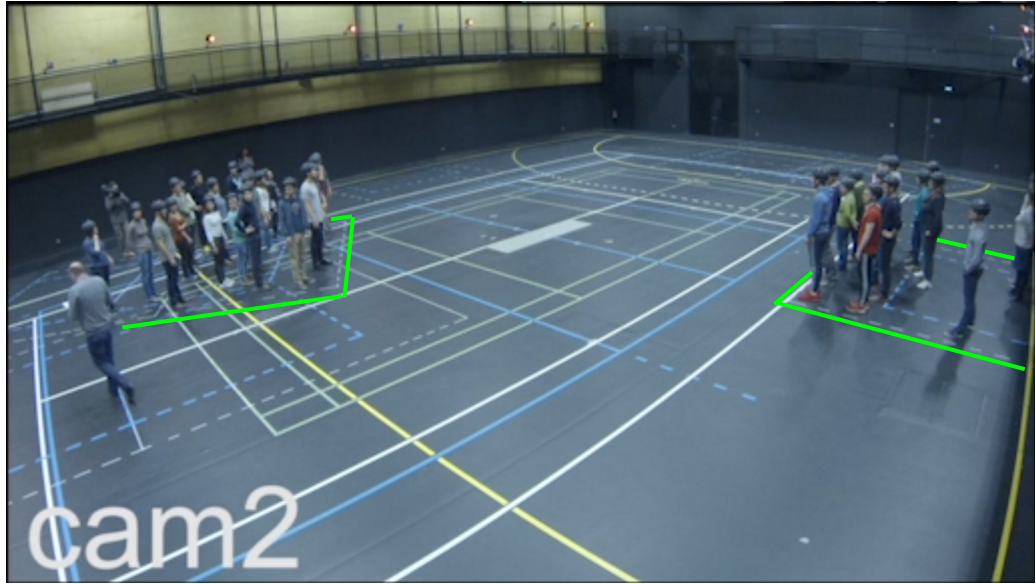

Supplement: S7 Fig — This figure is basically the snapshot of video in the next item at time 00:00. Before the onset of the trial, the participants are seen to wait within the dashed squares, which are clearly pointed out by light-green lines. (PDF) [file pcbi.1010210.s009.pdf]
