# Supplementary material for: Analysis of emergent patterns in crossing flows of pedestrians reveals an invariant of ‘stripe’ formation in human data
Source: PLoS Comput Biol. 2022 Jun 9;18(6):e1010210. doi: 10.1371/journal.pcbi.1010210 (PMC9216623; doi:10.1371/journal.pcbi.1010210)
Supplement: S1 Document — (PDF) [file pcbi.1010210.s015.pdf]

INVENTEURS DU MONDE NUMÉRIQUE

Le Président-directeur général

Date : 16 juin 2016

Auteur : Le Président-directeur général

Objet :

Avis du COERLE sur les expérimentations intitulées « Interactions entre marcheurs»

Nature : Information et action

Destinataires : Stéphane UBEDA, Julien PETTRÉ, Anne-Hélène OLIVIER, Ludovic ME

Copies : membres du COERLE, correspondants COERLE, Didier BENZA

Madame, Monsieur,

Vous trouverez en pièce jointe l'avis du COERLE n° 2016-008 concernant la demande citée en objet.

Cordialement,

Le Président-directeur général

Antoine Petit

INVENTEURS DU MONDE NUMÉRIQUE

COERLE
COMITE OPERATIONNEL D'EVALUATION
DES RISQUES LEGAUX ET ETHIQUES

Rocquencourt, le 15 juin 2016

AVIS n°2016-008

du Comité Opérationnel d'Evaluation des Risques Légaux et Ethiques (COERLE)

Le présent avis est rendu suite à la saisine du COERLE faite le 3 juin 2016 par Stéphane Ubeda, directeur du Centre de Rennes - Bretagne Atlantique.

Il porte sur l'étude des déplacements de groupes de personnes et du comportement collectif qui émerge de l'interaction entre les individus de ces groupes.

Le projet s'intitule : «Interactions entre marcheurs»

Les porteurs sont :

- Julien PETTRÉ, chargé de recherche Inria dans l'équipe-projet MimeTIC du Centre de Rennes - Bretagne Atlantique.
- Anne-Hélène OLIVIER, maître de conférence à l'université de Rennes 2, membre de l'équipe-projet MimeTIC

en collaboration avec :

- William WARREN, professeur à Brown University, USA.

L'avis du COERLE est sollicité pour valider les aspects légaux et éthiques des expérimentations et leurs contextes ainsi que pour permettre le cas échéant la publication de tout ou partie des résultats.

Pour formuler son avis, le COERLE a disposé du dossier de demande d'autorisation et de plusieurs interactions avec les porteurs.

Contexte :

Lors du déplacement d'un groupe de 40 personnes, l'objectif de l'étude est de comprendre comment un comportement collectif émerge des interactions locales entre les membres du groupe.
Ce projet se déroule dans le cadre de l'équipe-projet MimeTIC commune entre Inria et les Universités de Rennes 1 et 2 ainsi que l'ENS Bretagne. Il est additionnement financé par le projet ANR Percolation et SIMS, équipe associée avec l'université de Brown.

Les expérimentations consisteront en des séances de 2h au cours desquelles, un groupe de 40 personnes sera amené à se déplacer dans le gymnase de l'ENS à Rennes.
Pour organiser chaque expérimentation, les porteurs seront assistés de plusieurs membres de l'équipe-projet MimeTIC ainsi que d'étudiants en thèse dans le laboratoire M2S ou de l'université de Brown.
Chaque expérimentation sera filmée.

Domaine de Voluceau - Roquencourt B.P. 105
78153 Le Chesnay Cedex France
Tél. : +33 (0)1 39 63 55 11
Fax : +33 (0)1 39 63 55 30

www.inria.fr

Remarques du COERLE :

A. Dans le cadre de l'équipe-projet MimeTIC l'expérimentation est bien motivée ;
B. La saisine est bien rédigée et décrit les objectifs et procédures ;
C. Les porteurs des expériences sont expérimentés et formés à l'éthique de ce type de travaux ;
D. Les données personnelles (contact, genre, âge, taille) des participants sont enregistrées et sont utiles à l'exploitation scientifique de l'expérience ;
E. Le correspondant informatique et liberté devra être contacté et informé des données personnelles gérées : son accord explicite est nécessaire ;
F. Le protocole d'utilisation des enregistrements vidéos est bien décrit dans la saisine et en particulier conduira à flouter les visages en cas d'utilisation dans des publications ;
G. Les participants seront des personnes majeures bien portantes ;
H. Le formulaire de consentement ne mentionne pas les mêmes porteurs du projet que la saisine : à rectifier ;
I. Les données qui sont décrites comme collectées dans le formulaire de consentement éclairé ne sont pas les mêmes que dans la saisine : à homogénéiser ;
J. Le floutage des photos ou des vidéos qui est mentionné dans la saisine n'est pas mentionné dans le consentement éclairé : le statut des vidéos et des photos devra être rendu globalement cohérent ;
K. La protection des données est bien décrite ;
L. La durée de conservation des données devra être précisée et les personnes en charge de leur traitement devront être nommément désignées. En particulier si les données sont conservées au delà de la durée du projet (ce qui peut faire sens pour des raisons de reproductibilité scientifique), préciser avec quel but, comment et où. Si des données doivent être effacées préciser dans quelles conditions et qui sera en charge de le réaliser.
M. Préciser la démarche prévue par les expérimentateurs en cas de découverte inopinée ;
N. Les porteurs du projet devront s'assurer de la formation suffisante en éthique des expérimentateurs impliqués dans la conduite de l'expérimentation ;
O. Pour les prochaines saisines, les porteurs devront prévoir un délai plus important entre le dépôt de la saisine et le début éventuel des expérimentations.

Avis du COERLE :

Le COERLE donne un avis favorable à la mise en place de cette expérience sous réserve de la prise en compte des remarques suivantes :

- Les dates de début et de fin devront être notifiées au COERLE ;
- Les éléments complémentaires concernant les remarques ci-dessus doivent être fournies au COERLE sous un mois.

A des fins de suivi, un court rapport (une page maximum) fera le point sur le déroulement de l'expérience au plus tard 6 mois après son début.

Le président du COERLE

Claude KIRCHNER

Domaine de Voluceau - Roquencourt B.P. 105
78153 Le Chesnay Cedex France
Tél. : +33 (0)1 39 63 55 11
Fax : +33 (0)1 39 63 55 30

www.inria.fr
